# Governance for health: the HIV response and general global health

**DOI:** 10.2471/BLT.19.230417

**Published:** 2019-03-01

**Authors:** Linda-Gail Bekker, Jirair Ratevosian, Julia Spencer, Peter Piot, Chris Beyrer

**Affiliations:** a The Desmond Tutu HIV Centre, Cape Town, South Africa.; b Department of Epidemiology, Johns Hopkins University, 615 N. Wolfe Street Suite E7152, Baltimore, Maryland 21205, United States of America (USA).; c London School of Hygiene & Tropical Medicine, London, England.; d Centre for Public Health and Human Rights, Johns Hopkins University, Baltimore, USA.

In the editorial *Shifting global health governance towards the sustainable development goals*,^1^ Marten and colleagues argue that the *Transforming our world: the 2030 agenda for sustainable development* offers an important opportunity to broaden the scope of global health governance. The sustainable development goals (SDGs) mark an expansion from the millennium development goals (MDGs) in scope and ambition, and recognize good health and wellbeing as a broad development issue.^2^ However, Marten et al. argue that the expanded health scope of the SDGs has not been met with any notable reforms to global health governance, with the current architecture still mostly intended to address the MDGs, not the SDGs.^1^

We agree that achieving the SDGs and starting a new era of global health solidarity will require a paradigm shift in global health governance,^3^ moving beyond silos, rethinking current institutions and developing better coordinating approaches to achieve health targets. Such a shift took place with the global response to the acquired immunodeficiency syndrome (AIDS), which altered global health governance on an unprecedented scale.^4^ The response showed what is possible when activism, political leadership, and science and community-driven responses come together to challenge the status quo.^4^ Yet even these gains are fragile. The recent report of the International AIDS Society — Lancet Commission shows that we are not on track to end AIDS (SDG 3.3) and calls for efforts to rejuvenate the global response. The risk of not doing so is a resurgence of the human immunodeficiency virus (HIV) epidemic. At the same time, the commission demonstrates that immediate action is needed to link and synergize HIV services with other health services.^5^

The commission modelled potential outcomes of carefully integrating HIV and other health services in India, Kenya, Nigeria, the Russian Federation^6^ and South Africa, and showed that this integration can be cost–effective for improving HIV and broader health outcomes.^5^ In Kenya, for instance, introducing a mobile screening programme for HIV, diabetes and hypertension over 10 years would identify 686 000 individuals with untreated diabetes and 7.57 million people with untreated hypertension. The same screening programme is projected to result in a 44% decline in new HIV infections by 2028, that is, 216 655 new HIV infections and 244 374 AIDS-related deaths averted.^5^ Similarly, in Nigeria, integrating reproductive health and HIV services over a period of 10 years was shown to avert more than 8 million unintended pregnancies and to reduce the number of infants acquiring HIV by 56%, preventing more than 237 500 babies acquiring HIV by 2028.^5^ These modelling scenarios suggest that by working closely together, the HIV and wider global health community can accelerate progress towards achieving the SDGs; however, this approach will require transformed governance and financing mechanisms that take into account diverse contexts and health needs.

In developing modern governance mechanisms for the future, the robust systems developed to respond to the HIV epidemic over the last three decades can be a starting point. Yet, as donors manage transitions of health programmes to middle-income countries to ensure ownership, there is serious risk of diminished funding for key populations that are particularly vulnerable to HIV.^7^ The United States President’s Emergency Plan for AIDS relief and The Global Fund to Fight AIDS, Tuberculosis and Malaria must adapt to ensure that transition metrics are developed and implemented. Donors must develop new diplomacy strategies and support regional mechanisms to complement aid transitions and hold countries accountable for protecting rights and ensuring adequate health financing and programming for all their citizens.

We must also preserve key attributes of the AIDS response that have been critical to success, from the unwavering commitment to human rights and gender equity to mobilizing a truly multisectoral response grounded in principles of inclusivity, transparency and accountability, with the full participation of affected communities and civil society.^5^ However, even the most enlightened global response mechanism must adapt to remain efficient, accountable and responsive. Over the last months, much has been said about the governance crisis at the Joint United Nations Programme on HIV/AIDS (UNAIDS)^8^ and its future.^9^^–^^12^ These challenging times call for difficult choices and new ideas to transform UNAIDS and preserve the very core strengths of the HIV response. Achieving the SDGs will require transformed governance efforts to shift the focus upstream on the structural determinants of health and new leverage mechanisms to fit economic and geopolitical trends. Implementing an integrated governance system will inevitably be complex and require an honest consideration of the strengths and weaknesses of the actors involved in health governance.^5^ To this end, the commission will remain active in the pursuit of effective global health governance strategies that are needed to drive health solidarity and revitalize the global response to HIV.
